# Modeling of Singlet Oxygen Generation and Thermal Effects During Laser–Tissue Interaction

**DOI:** 10.3390/ma18214908

**Published:** 2025-10-27

**Authors:** Marek Jasiński, Maria Zadoń

**Affiliations:** Department of Computational Mechanics and Engineering, Silesian University of Technology, Konarskiego 18A, 44-100 Gliwice, Poland; marek.jasinski@polsl.pl

**Keywords:** bioheat transfer, singlet oxygen generation, laser–tissue interactions, Krogh cylinder model, boundary element method, finite difference method, shooting method

## Abstract

This paper presents an analysis of the phenomena that occur during photodynamic therapy (PDT). For this purpose, models of laser energy deposition, bioheat transfer, and reactions occurring during the PDT process were used. Light distribution was estimated on the base of optical diffusion equation, while for the bioheat analysis the Pennes formula has been used. The PDT reaction model includes equations related to the concentration of triplet oxygen, photosensitizer, and singlet oxygen. The tissue perfusion coefficient and the effective scattering coefficient have been assumed to be thermally damage dependent. Changes in blood velocity in capillary, which affects maximum oxygen supply in PDT model, were also considered. A way of modeling the abnormal vascular pattern in the tumor area was also proposed, and the initial distribution of triplet oxygen in the tumor region was determined on the Krogh cylinder model. At the stage of numerical calculation, the boundary element method, the finite difference method, and the shooting method were used.

## 1. Introduction

There is no need to convince anyone that various types of cancer are the bane of the modern world. Of course, as technology advances, newer and newer cancer therapies are available, and they are being refined even further over time. However, the diversity of cancer tissues means that in many situations, the final outcomes of therapy do not meet expectations. Tumor tissues differ in many features from healthy tissues, and one of such features is the irregular arrangement of the vascular network. This means that the capillaries that are responsible for delivering oxygen to tissues can have unusual shapes in addition to being chaotically organized. Instead of the typical shape of straight tubes, they can be in the form of bulbs, be tortuous, or simply blindly end. This type of structure leads to microthrombosis, resulting in hypoxia and deficiency of oxygen within the tumor [1,2,3].

One of the widely used methods in the treatment of cancer is photodynamic therapy (PDT), which is based on chemical reactions between oxygen in the tissue, triplet oxygen (^3^O_2_), and a special substance, the photosensitizer (S_0_), which is administered to the patient. Then, as a result of light exposure to the tissue, ^3^O_2_ is excited, resulting in its conversion to the singlet oxygen form (^1^O_2_), which is highly cytotoxic to cancer cells. This is known as Type II reactions, while in reactions known as Type I, direct interaction of the excited photosensitizer is utilized. It is clear that since the abnormal vascular pattern mentioned earlier affects oxygen delivery to the tumor area, it can also affect the final outcome of PDT treatment [1,2,3,4,5,6,7].

Light, such as in the form of a laser beam acting on biological tissue, can cause a local increase in its temperature, which in turn leads to alteration of tissue parameters. On the other hand, the temperature increases achieved during PDT are generally not large (with the exception of PDT varieties in which temperature is used as an additional therapeutic agent), so they do not cause permanent thermal damage to the tissue [1,2]. One of the parameters of biological tissue is the perfusion coefficient, the value of which is altered with a change in temperature (e.g., increases, as a result of blood vessels vasodilation with a slight temperature increase), which can also ultimately affect the oxygen concentration during the PDT procedure [8,9,10,11].

The model which is considered in current work takes into account the photothermal and photochemical phenomena in the tissue during PDT. The former are the result of laser–tissue interactions, while the latter are related to reactions occurring during the generation of singlet oxygen in the tissue. In addition, a method to account for the irregular distribution of oxygen in the tumor area resulting from its irregular structure and the other characteristics of this type of tissue described above is presented. To the best of the author’s knowledge, apart from his earlier work, papers in which bioheat transfer, PDT reactions, and oxygen distribution models are considered together are not present in the literature, so it can be said that this fills some kind of gap.

The basic oxygen distribution model is the so-called Krogh cylinder, which represents the cylindrical tissue area around the capillary. On the mathematical side, it contains differential equations for the subdomains of tissue and capillary, where the dependent variable is the oxygen partial pressure. These equations must be supplemented by equations related to hemoglobin saturation in the capillary subdomain, and the essential connecting element is the adopted model of the oxygen dissociation curve (ODC) [11,12,13,14,15,16]. It should be noted that the need to adopt the ODC model is due to the presence of two forms of oxygen in the blood: free molecules and chemical bonds that form oxyhemoglobin. The first is described by partial oxygen pressure. Oxyhemoglobin releases oxygen when the partial pressure decreases, and reverse reactions are also possible. The Krogh cylinder model was formulated in 1919, with numerous simplifications at its base. However, over time, it became the basis for many works related, among other things, to the process of angiogenesis, the presence of tumor cells in tissue, or various therapies [4,5,11,12,17].

The full mathematical description of the processes that occur during PDT is quite complicated, so a simplified model containing reaction equations for the three main PDT agents is most often used: triplet oxygen, singlet oxygen, and photosensitizer. Phenomena accompanying the reactions such as absorption, fluorescence, energy transfer, and photobleaching are included in the model through a set of photochemical parameters typical for the photosensitizer under consideration. The PDT model is mainly dedicated to working to improve the quality of the final treatment effects, mainly associated with the level of singlet oxygen that guarantees the overall effectiveness of the therapy [4,5,10].

The Pennes equation is the oldest, but still probably the most common bioheat transfer equation. In general, it is based on the classical Fourier law of conduction, in which the presence of small blood vessels and metabolic phenomena is taken into account through appropriate source heat functions [10,13,18,19]. Equations such as Cattaneo–Vernotte (hyperbolic thermal wave model) or dual-phase lag allow for taking into account the delay of the heat flux with respect to the temperature gradient (relaxation time) and the delay in gradient of temperature caused by heat transfer in microscale structures (thermalization time). Each of these equations is widely discussed in the literature [20,21,22,23,24,25,26,27,28,29,30,31,32,33].

Knowledge of laser energy deposition is essential to account for local temperature increases in the tissue through the appropriate source function in the adopted bioheat transfer model and for the reaction model during PDT. Various models are possible, generally based on the radiative transport equation, which is due to the fact that for soft tissues for wavelengths between 650 and 1300 nm the scattering generally dominates over the absorption. Both statistical Monte Carlo methods and several modifications of the discrete ordinates method are used to solve the radiative transport equation [20,25,29,34,35,36,37,38].

In summary, the work deals with the analysis of phenomena during PDT treatment, taking into account the laser energy deposition model, bioheat transfer model, PDT reactions model and Krogh cylinder model. The description of light energy distribution in the tissue is based on the optical diffusion equation. The Pennes equation is used in bioheat transfer analysis. The perfusion coefficient and effective scattering coefficient are considered as dependent on tissue thermal damage. In turn, the blood velocity in the capillaries is assumed to be dependent on perfusion with an influence on the value of the maximum oxygen supply rate in the PDT model and initial triplet oxygen concentration obtained on the basis of the Krogh cylinder model. In addition, a coefficient is included reflecting the tortuosity of capillaries in the tumor subdomain. In the PDT reactions model, concentration equations for triplet oxygen, singlet oxygen, and photosensitizer are taken into account. The tumor subdomain is distinguished by adopting a vascular pattern model. In the stage of numerical implementation, the boundary element method (bioheat transfer analysis), the finite difference method (PDT model and laser energy deposition model), and the shooting method (Krogh cylinder model) are used.

## 2. Materials and Methods

### 2.1. Governing Equations

**Figure 1:**
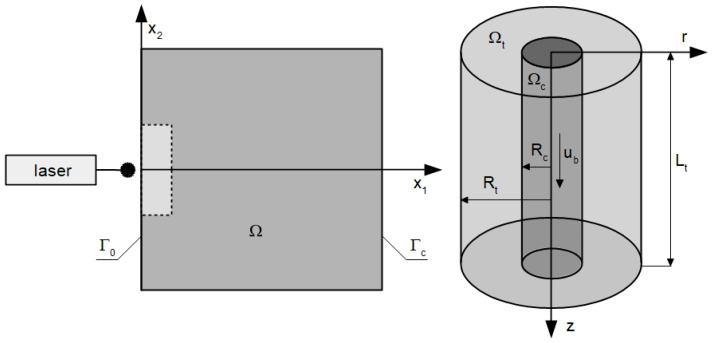
Domains considered in parts in bioheat transfer and PDT problems (**left**) and model used for determination of initial concentration of triplet oxygen (**right**).

In the current work, the 2D domain of biological tissue subjected to laser action is considered (Figure 1 left). The tissue is treated as a homogeneous domain in the bioheat transfer part of the analysis, and the tumor subdomain marked by the dashed line and brighter rectangle is distinguished only in the part related to the analysis of photochemical reactions occurring during the PDT treatment. Figure 1 right shows the Krogh cylinder, the axisymmetric model that was used to determine the initial concentrations of triplet oxygen in tissue.

The model consists of a set of differential reaction equations for three main factors in the PDT process, that is, triplet oxygen (^3^O_2_), singlet oxygen (^1^O_2_), and a photosensitizer (S_0_) [4,5,10]:

(1) $$\begin{array}{ll} x\in \Omega : & \left\{\begin{array}{ll} \frac{dc_{3O2}}{dt}+\gamma c_{S0}=\psi_{sup}, & \gamma =\frac{\xi \phi c_{3O2}}{c_{3O2}+\beta }\\ \frac{dc_{S0}}{dt}+\gamma \sigma c_{S0}\left(c_{S0}+\delta \right)=0, & \psi_{sup}=\psi_{sup,max}\left(1-\frac{c_{3O2}}{c_{3O2,init}}\right)\\ \frac{dc_{1O2}}{dt}-\gamma c_{S0}=0 & \end{array}\right.\\ t=0: & c_{3O2}=c_{3O2,init},\;c_{S0}=c_{S0,init},\;c_{1O2}=0 \end{array}$$

where *c*_3O2_, *c*_S0_, *c*_1O2_ [mol cm^−3^] are the concentrations of triplet oxygen, sensitizer, and singlet oxygen, respectively; parameters β [mol cm^−3^], σ [cm^3^ mol^−1^], ξ [cm^2^ mW^−1^ s^−1^] and δ [mol cm^−3^] are the PDT photochemical parameters defined as the oxygen quenching threshold concentration, the specific photobleaching ratio, the specific oxygen consumption rate, and the low concentration correction term, respectively; ψ*_sup_* [mol cm^−3^ s^−1^] is the oxygen supply rate; and ψ*_sup,max_* [mol cm^−3^ s^−1^] is the maximum oxygen supply rate.

To induce the reactions described by Equation (1), it is necessary to determine the total fluence rate ϕ [mW cm^−2^] resulting from solving the task related to the deposition of laser energy in tissue. It should be noted that the total fluence rate is the sum of collimated ϕ*_c_* and diffused ϕ*_d_* parts. Due to domination of ϕ*_d_* for most soft tissues, the value of ϕ*_c_* is sometimes neglected, especially in singlet oxygen generation models. Because in this work both photochemical and photothermal effects are considered, both components of the total fluence rate were taken into account [1,4,9,10].

The collimated part of the fluence rate ϕ*_c_* is determined by the Beer–Lambert law of absorption [1,10,33]:

(2) $$\phi_{c}(x)=\phi_{0}\exp\left(-\frac{2x_{2}^{2}}{r_{beam}^{2}}\right)\exp(-{\mu^{\prime }}_{t}x_{1})$$

whilst the determination of the diffuse component ϕ*_d_* is based on the optical diffusion equation [4,9,33,34]:

(3) $$\begin{array}{ll} \begin{array}{c} x\in \Omega : \end{array} & \nabla \left[D\nabla \phi_{d}(x)\right]-\mu_{a}\phi_{d}(x)+{\mu^{\prime }}_{s}\phi_{c}(x)=0\\ x\in \Gamma_{0}\cup \Gamma_{c}: & -\nabla \left[D\phi_{d}(x)\right]\cdot n=\frac{1}{2}\phi_{d}(x)\\ & D=\frac{1}{3\left[\mu_{a}+\left(1-g\right)\mu_{s}\right]}=\frac{1}{3{\mu^{\prime }}_{t}}\\ & {\mu^{\prime }}_{t}=\mu_{a}+{\mu^{\prime }}_{s}=\mu_{a}+(1-g)\mu_{s} \end{array}$$

where μ*_a_*, μ*_s_*, μ*_t_* [cm^−1^] denote the absorption, scattering, and attenuation coefficients, respectively, *D* is the diffusion coefficient, *g* is the anisotropy coefficient, while ϕ_0_ [mW cm^−2^] is the surface irradiance of the laser. The values μ′*_s_* and μ′*_t_* are the effective scattering coefficient and the effective attenuation coefficient, and *r_beam_* is the radius of the laser beam.

The bioheat transfer analysis is based on the Pennes equation with appropriate boundary-initial conditions [8,13,18,39,40]:

(4) $$\begin{array}{ll} x\in \Omega : & c\frac{\partial T(x,t)}{\partial t}=\lambda \nabla^{2}T(x,t)+Q_{met}+Q_{perf}+Q_{las}\\ x\in \Gamma_{0}: & q(x,t)=\alpha \left[T(x,t)-T_{amb}\right]\\ x\in \Gamma_{c}: & q(x,t)=0\\ t=0: & T(x,t)=T_{init} \end{array}$$

where *c* [J m^−3^ K^−1^] is volumetric specific heat, λ [W m^−1^ K^−1^] is thermal conductivity, *T* denotes the temperature, *q* [W m^−2^] is the external heat flux, α [W m^−2^ K^−1^] is the convection coefficient, *T_amb_* is the ambient temperature while *T_init_* is the initial tissue temperature, Γ_0_ is the external boundary of the tissue, at which laser irradiation operates, and Γ*_c_* is the remaining part of the tissue boundary.

There are also three components in the Equation (4) related to the presence of internal heat sources associated with perfusion *Q_perf_*, metabolism *Q_met_*, and the impact of the laser on tissue *Q_las_* [W m^−3^]. The metabolic heat source *Q_met_* is considered a constant value, while the other two components are defined as [1,18,19,29]:

(5) $$\begin{array}{cc} Q_{perf}(x,t)=c_{B}w\left[T_{B}-T(x,t)\right] & Q_{las}(x,t)=\mu_{a\;}\phi (x) \end{array}$$

where *w* [s^−1^] is the perfusion coefficient, *c_B_* [J m^−3^ K^−1^] is the volumetric specific heat of the blood, and *T_b_* is the arterial temperature. It can be noticed that the total fluence rate ϕ, calculated in the laser energy deposition task, is taken into account in *Q_las_*.

In the current work, two tissue parameters, that is, the effective scattering coefficient and the perfusion coefficient, were also assumed to depend on thermal damage to the tissue [8,10,17,40,41,42]:

(6) $${\mu^{\prime }}_{s}(Arr)={\mu^{\prime }}_{s\;nat}\exp(-Arr)+{\mu^{\prime }}_{s\;den}[1-\exp(-Arr)]$$

and

(7) $$w=w(Arr)=\left\{\begin{array}{ll} \left(1+25Arr-260Arr^{2}\right)w_{0}, & 0\leq Arr\leq 0.1\\ \left(1-Arr\right)w_{0}, & 0.1<Arr\leq 1\\ 0, & Arr>1 \end{array}\right.$$

where *Arr* is the Arrhenius integral, on the basis of which the degree of tissue thermal damage can be estimated [8,10,17,29]:

(8) $$Arr(x,t^{F})=\int_{0}^{t^{F}}A\exp\left[-\frac{E}{RT(x,t)}\right]dt$$

In the above formulas, *w*_0_ is the initial perfusion coefficient, while μ′*_s nat_* and μ′*_s den_* are effective scattering coefficients for native and thermally damaged tissue, *E* [J mol^−1^] is the activation energy, *A* [s^−1^] is the preexponential factor, and *R* [J mol^−1^ K^−1^] is the universal gas constant.

It should be clarified that the Functions (6) and (7) were adopted because they capture quite well the effects occurring during the tissue heating. The function of the effective scattering coefficient captures the increase in the value of this coefficient, with an increase in thermal damage, which is often associated with the apparent effect of tissue bleaching. On the other hand, the function for the perfusion coefficient takes into account its initial increase caused by vasodilatation (range [0, 0.1]) and subsequent decrease in blood flow resulting from damage to the vasculature damage (range (0.1, 1]). However, since the temperature increases achieved during PDT are not large, it is not expected to reach the threshold *Arr* = 1*,* indicating full tissue damage [1,8,24,43].

The basis for the connection of bioheat transfer and PDT models is the perfusion coefficient. It represents the presence of blood in the tissue on a macro scale, taking into account the volume and velocity of blood in all blood vessels located in the tissue. Assuming, according to the assumptions of the Krogh model, that the tissue consists of identical adjacent cylindrical regions of tissue containing capillary, it can be written [16,43]:

(9) $$w=\frac{Q_{b}}{\pi R_{t}^{2}L_{t}}=\frac{\pi R_{c}^{2}u_{b}}{\pi R_{t}^{2}L_{t}}$$

where *R_c_* [μm] is the capillary radius, *R_t_* [μm] is the tissue cylinder around capillary radius, and *L_t_* [μm] is the tissue cylinder length, while *u_b_* [cm s^−1^] and *Q_b_* [cm^3^ s^−1^] denote the blood velocity in the capillary and blood flow rate in the capillary, respectively.

In the current work, besides variable perfusion coefficient (Equation (7)), an additional coefficient *m_tort_* was introduced that denotes the tortuosity of the capillary. For healthy tissue, the coefficient is always equal to 1, while for tumor, it is random from the interval (0, 1]. Given these assumptions and the Formula (9), the blood velocity in the capillaries is of the form:

(10) $$u_{b}=m_{tort}w(Arr)L_{t}\frac{R_{t}^{2}}{R_{c}^{2}}$$

The calculated value of *u_b_* is necessary to determine the maximum supply rate ψ*_sup,max_*, the component of the PDT model (cf. Equation (1)), based on the formula [5,10]:

(11) $$\psi_{sup,max}=\frac{1200u_{b}R_{c}}{L_{t}{\left(R_{t}+b\right)}^{2}}\left(R_{c}+\frac{a^{2}+M_{0}^{2}}{2500-M_{0}^{2}}\right)$$

where *M*_0_ [mol cm^−3^ s^−1^] is the oxygen consumption rate, while *a* and *b* are the coefficients depending on the type of tissue (healthy or tumorous).

In order to determine the initial concentration of triplet oxygen in the tissue, the axisymmetric Krogh cylinder model shown in Figure 1 right was used. A mathematical description was adopted in the form of two equations for the radial and axial directions. For the radial direction, one has [13,16,17,41,43]:

(12) $$\begin{array}{ll} r\in \Omega_{t}: & K_{t}\nabla^{2}P_{t}=\frac{M_{0}P_{t}}{P_{crit}+P_{t}}\\ r=R_{c}: & 2\pi R_{c}K_{t}\nabla P_{t}=-k\left(P_{b}-P_{t}\right)\\ r=R_{t}: & \nabla P_{t}=0 \end{array}$$

where *P_t_* and *P_b_* [mmHg] are the partial oxygen pressure in tissue and blood, respectively, *K_t_* [(cm^2^ s^−1^)(mol cm^−3^ mmHg^−1^)] is the Krogh diffusion coefficient, *P_crit_* [mmHg] is the partial pressure corresponding to half maximum oxygen consumption, and *k* [(cm^2^ s^−1^)(mol cm^−3^ mmHg^−1^)] is the mass transfer coefficient.

The partial pressure of oxygen in the capillary *P_b_* changes along the Krogh cylinder, which is expressed as [16,41,43]:

(13) $$\begin{array}{ll} z\in \Omega_{c}: & \pi R_{c}^{2}u_{b}\kappa_{b}\nabla S_{Hb}=-k\left(P_{b}-P_{t}\right)\\ z=0: & P_{b}=P_{b\;inlet} \end{array}$$

where *u_b_* [μm s^−1^] denotes the blood velocity in capillary expressed by Equation (10), κ*_b_* [mol cm^−3^_blood_] is the oxygen carrying a capacity of blood at 100% saturation, while *S_Hb_* is the saturation of oxyhemoglobin. After the *S_Hb_* value is determined, the partial oxygen pressure in the capillary is determined using the inverted oxyhemoglobin dissociation curve (ODC). In this work, the Hill model of ODC is assumed in the form [5,11,12,13,14,16]:

(14) $$\begin{array}{ccc} S_{Hb}=\frac{P_{b}^{n}}{P_{b}^{n}+P_{50}^{n}} & \to & P_{b}=P_{50}{\left(\frac{S_{Hb}}{1-S_{Hb}}\right)}^{\frac{1}{n}} \end{array}$$

where *n* is the Hill coefficient while *P*_50_ denotes the oxygen pressure corresponding to 50% hemoglobin saturation.

Finally, after determining the partial oxygen pressure distribution (Equations (12)–(14)), the initial concentration of triplet oxygen is estimated on the basis of the formula:

(15) $$c_{3O2,init}=\frac{\alpha_{3O2,t}}{\left(R_{t}-R_{c}\right)L_{t}}\int_{0}^{L_{t}}\int_{R_{c}}^{R_{t}}P_{t}drdz$$

where *α*_3O2,*t*_ [mol cm^−3^ mmHg] is the solubility of oxygen in the tissue [5,11].

It should be clarified that in the cases of analysis of oxygen distribution in biological tissue using the Krogh cylinder, single cylinders are usually considered, since the partial pressure values determined from them are intended to represent the average conditions of the tissue (in accordance with the aforementioned assumption of the Krogh model that the tissue consists of adjacent cylinders) [11,14,16]. However, since this work considers a tumor subdomain with an abnormal vascular pattern and thus heterogeneous oxygen distribution, some number of Krogh cylinders will be considered, with different capillary dimensions and different values of blood velocity and capillary *u_b_* and the oxygen consumption rate *M*_0_. Detailed information on how to adopt the model of abnormal vascular pattern is presented in the Section 3.

**Figure 2:**
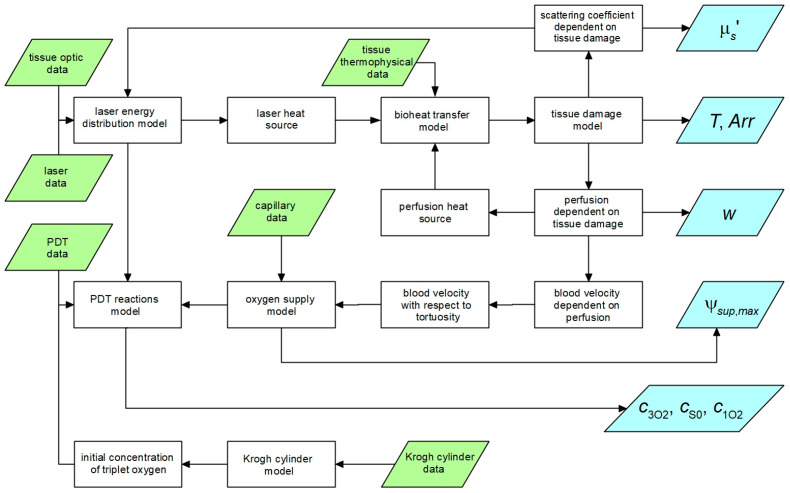
Data flow between particular parts of the simulation.

The flow of the data between particular parts of the simulation is illustrated in Figure 2 (trapezoids are input data and the results).

### 2.2. Methods of Solution

The finite difference method was used to solve the set of coupled equations included in the PDT model (1). Differential quotients were substituted in place of time derivatives, which, after appropriate transformations, gave the following differential equations [9,39,40]:

(16) $$\begin{array}{l} c_{3O2,i}^{f}=c_{3O2,i}^{f-1}-\Delta t\left[\frac{\xi \phi c_{S0,i}^{f-1}c_{3O2,i}^{f-1}}{c_{3O2,i}^{f-1}+\beta }-\psi_{sup,max}\left(1-\frac{c_{3O2,i}^{f-1}}{c_{3O2,init}}\right)\right]\\ c_{S0,i}^{f}=c_{S0,i}^{f-1}-\Delta t\frac{\sigma \xi \phi c_{S0,i}^{f-1}c_{3O2,i}^{f-1}\left(c_{S0,i}^{f-1}+\delta \right)}{c_{3O2,i}^{f-1}+\beta }\\ c_{1O2,i}^{f}=c_{1O2,i}^{f-1}+\Delta t\frac{\xi \phi c_{S0,i}^{f-1}c_{3O2,i}^{f-1}}{c_{3O2,i}^{f-1}+\beta } \end{array}$$

**Figure 3:**
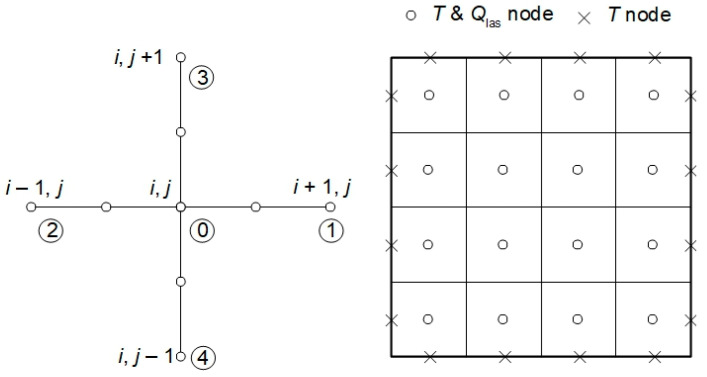
Five-point stencil used in the laser energy deposition task and discretization used in the bioheat transfer problem.

The finite difference formulas were also used to solve a laser energy deposition in tissue task (3). The assumed differential quotients for the stencil presented in Figure 3 are of the form (*l* denotes the grid step) [10,43]:

(17) $$\begin{array}{cc} {\left(D\frac{\partial \phi_{d}}{\partial x_{1}}\right)}_{i+0.5,j}=D_{01}\frac{\phi_{d\;1}-\phi_{d\;0}}{l}, & {\left(D\frac{\partial \phi_{d}}{\partial x_{1}}\right)}_{i-0.5,j}=D_{02}\frac{\phi_{d\;0}-\phi_{d\;2}}{l}\\ {\left(D\frac{\partial \phi_{d}}{\partial x_{2}}\right)}_{i,j+0.5}=D_{03}\frac{\phi_{d\;3}-\phi_{d\;0}}{l}, & {\left(D\frac{\partial \phi_{d}}{\partial x_{2}}\right)}_{i,j-0.5}=D_{04}\frac{\phi_{d\;0}-\phi_{d\;4}}{l} \end{array}$$

where *D*_0*e*_ = 2*D*_0_*D_e_*/(*D*_0_ + *D_e_*), so, the left-hand side operator of (3), for the central node, has the form:

(18) $${\left[\frac{\partial }{\partial x_{1}}\left(D\frac{\partial \phi_{d}}{\partial x_{1}}\right)+\frac{\partial }{\partial x_{2}}\left(D\frac{\partial \phi_{d}}{\partial x_{2}}\right)\right]}_{i,j}=\frac{1}{l}\sum_{e=1}^{4}D_{0e}\left(\phi_{d\;e}-\phi_{d\;0}\right)$$

The final equation for the central node of the stencil is as follows:

(19) $$\phi_{d\;0}=\left(\sum_{e=1}^{4}D_{0e}\left(\phi_{d\;e}-\phi_{d\;0}\right)+l{\mu^{\prime }}_{s}\phi_{c\;0}\right)/\left(\sum_{e=1}^{4}D_{0e}+l\mu_{a}\right)$$

In order to solve the bioheat transfer problem (4), the first scheme of boundary element methods was applied. For this variant of BEM, in the 2D problem, for transition *t^f^*^−1^ → *t^f^* with constant time step Δ*t*, the boundary integral equation is of the form [44,45]:

(20) $$\begin{array}{c} B(\zeta )T(x,t^{f})+\int_{\Gamma }q(x,t^{f})g(\zeta ,x)d\Gamma =\int_{\Gamma }T(x,t^{f})h(\zeta ,x)d\Gamma \\ +\iint_{\Omega }q^{*}(\zeta ,x,t^{f},t^{f-1})T(x,t^{f-1})d\Omega +\iint_{\Omega }Q_{V}(x,t^{f-1})g(\zeta ,x)d\Omega \end{array}$$

where *Q_V_* is the sum of the internal heat function arising from perfusion, metabolism, and laser irradiation (cf. Equation (4)); T* and q* denote the fundamental solution and the heat flux resulting from the fundamental solution [10]:

(21) $$\begin{array}{cc} T^{*}(\zeta ,x,t^{f},t)=\frac{1}{4\pi a(t^{f}-t)}\exp\left[-\frac{r^{2}}{4a(t^{f}-t)}\right], & q^{*}=-\lambda \nabla T^{*}n \end{array}$$

where *r* is the distance between point under consideration **x** and the observation point ζ, *a* = λ/*c*, while *B*(ζ) is the coefficient from the interval (0, 1) and

(22) $$\begin{array}{cc} h(\zeta ,x)=\frac{1}{c}\int_{t^{f-1}}^{t^{f}}q^{*}(\zeta ,x,t^{f},t)dt, & g(\zeta ,x)=\frac{1}{c}\int_{t^{f-1}}^{t^{f}}T^{*}(\zeta ,x,t^{f},t)dt \end{array}$$

In the stage of numerical realization, the discrete form of (20) was used [45]:

(23) $$\sum_{j=1}^{N}G_{ij}q_{j}^{f}=\sum_{j=1}^{N}H_{ij}T_{j}^{f}+\sum_{l=1}^{L}P_{il}T_{l}^{f-1}+\sum_{l=1}^{L}Z_{il}{Q_{V}}_{l}^{f-1}$$

where *N* and *L* are the numbers of boundary and internal elements, respectively, while:

(24) $$\begin{array}{ll} G_{ij}=\int_{\Gamma_{j}}g(\zeta^{i},x)d\Gamma_{j}, & H_{ij}=\left\{\begin{array}{ll} \int_{\Gamma_{j}}h(\zeta^{i},x)d\Gamma_{j}, & i\neq j\\ -0.5, & i=j \end{array}\right.\\ P_{il}=\iint_{\Omega_{l}}T^{*}(\zeta^{i},x,t^{f},t^{f-1})d\Omega_{l}, & Z_{il}=\iint_{\Omega_{l}}g(\zeta^{i},x)d\Omega_{l} \end{array}$$

The “missing” boundary values of the temperatures and heat fluxes are calculated firstly, then, the temperatures at the internal points ζ*^i^* are estimated using the formula (*i* = *N* + 1, …, *N* + *L*)

(25) $$T_{i}^{f}=\sum_{j=1}^{N}H_{ij}T_{j}^{f}-\sum_{j=1}^{N}G_{ij}q_{j}^{f}+\sum_{l=1}^{L}P_{il}T_{l}^{f-1}+\sum_{l=1}^{L}Z_{il}{Q_{V}}_{l}^{f-1}$$

In the stage of solving the task of determining the partial oxygen pressure in the subdomain of tissue of the Krogh cylinder model (Equation (12)), shooting method was applied. In this method, a boundary value problem (BVP) is transformed into an initial value problem (IVP). The boundary condition on the selected boundary Γ*_shoot_* is used as the initial condition, while the second initial condition must be guessed. The problem defined in this way is then solved using one of the numerical methods for ODEs solution finding (in this work, the fourth-order Runge–Kutta method was used). The value of the solution value on the other boundary Γ*_target_* is compared with the boundary condition. If the difference between the value of the boundary condition and the solution obtained on the basis of IVP is unsatisfactory, the new initial value is guessed, and the problem is solved again. This procedure is repeated until the required agreement between the IVP solution and boundary condition on Γ*_target_* is obtained [41,46,47,48].

For BVP in the form of Equation (12), taking the boundary *r* = *R_t_* as Γ*_shoot_*, the IVP in cylindrical coordinates can be written as:

(26) $$\begin{array}{l} K_{t}{P_{t}}^{\prime \prime }+K_{t}\frac{1}{r}{P^{\prime }}_{t}=\frac{M_{0}P_{t}}{P_{crit}+P_{t}}\\ P_{t}(R_{t})=\alpha_{guess}\\ {P^{\prime }}_{t}(R_{t})=0 \end{array}$$

The following difference is checked after finding the solution of IVP:

(27) $$\begin{array}{l} \phi_{res}(R_{c},\alpha_{guess})={P^{\prime }}_{t}(R_{c},\alpha_{guess})-\frac{k}{2\pi R_{c}K_{t}}\left[P_{t}(R_{c},\alpha_{guess})-P_{b}\right] \end{array}$$

## 3. Results

The 2D domain of tissue with dimensions of 4 × 4 cm was analyzed in the current work. The tissue domain was treated as homogeneous in thermal analysis while in the part related to the PDT process, a tumor subdomain was also distinguished. The simulation parameters for the particular models are gathered in Table 1, Table 2 and Table 3.

The values of the photochemical parameters (δ, β, σ, ξ, Table 3) assumed in the PDT model correspond to the parameters of the photosensitizer Photofrin at 630 nm [5]. A 3600 s laser exposure was used for the analysis [5]. Additionally, the bioheat and PDT analyses require different value of time steps. For this reason, 1 s is assumed for bioheat task, and 0.1 s for PDT reactions model problem. The bioheats results from two consecutive time steps are linearly interpolated, and then we obtain the necessary values for the PDT task.

### 3.1. Vascular Pattern for Tumor Region

In the PDT-related part of the analysis, the tumor subdomain is distinguished. As already mentioned, capillaries in such an area are often irregularly arranged and have unusual shapes, which causes uneven oxygen distribution and areas of hypoxia. These features of the tumor tissue were taken into account by adopting different parameter values for the Krogh cylinder model, on the basis of which the initial concentration of triplet oxygen was determined.

**Figure 4:**
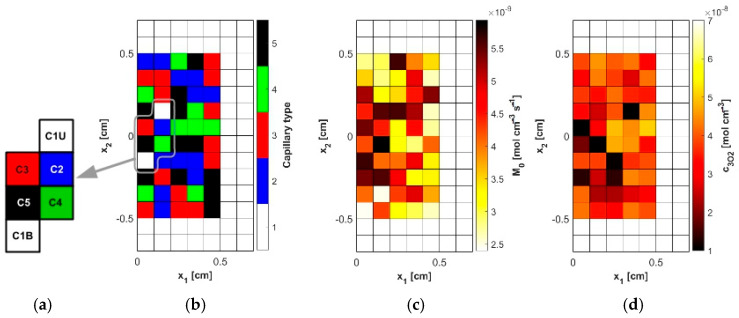
The model of the abnormal vascular pattern in tumor tissue ((**a**): selected capillaries, (**b**): random distribution of capillaries, (**c**): random distribution of the oxygen consumption rate *M*_0_, (**d**): calculated initial distribution of the triplet oxygen concentration *c*_3O2_).

**Figure 5:**
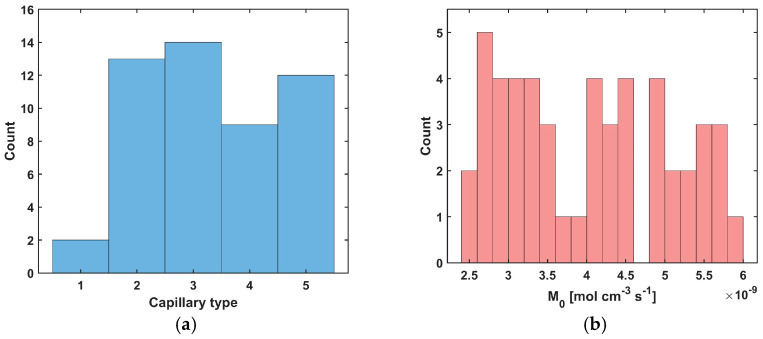
Histograms for random distribution of capillary type (**a**) and oxygen consumption rate *M*_0_ (**b**).

In the analysis, capillaries of five types, designated C1–C5 (Table 4), were assumed to be present. Capillary C1 was assumed to be typical for healthy tissue, while in the tumor subdomain, the capillary distribution was randomly generated (MATLAB R2024b Mersenne Twister generator with seed 0 was used, Figure 4b and Figure 5a), using the discretization introduced for the bioheat transfer problem solved by BEM (that is, the capillary types were linked to the internal elements of the BEM discretization). Randomly in the tumor domain, the oxygen consumption rate was also assigned as a value in the range *M*_0_ = 2.4 × 10^−9^ ÷ 6 × 10^−9^ [mol cm^−3^ s^−1^] (Figure 4c and Figure 5b). Since the oxygen demand, and thus the oxygen consumption, in the tumor tissue subdomain is generally higher than in healthy tissue, the lower limit of the range corresponds to the *M*_0_ value for healthy tissue. Furthermore, according to the data in [5], the Formula (11) was defined for healthy tissue, for which *a* = 100, *b* = 4.2, while for cancer tissue *a* = 50, *b* = −4.2. We note that *a* and *b* are introduced in Equation (11) as dimensionless coefficients, consistent with the original formulation [5]. The correctness of this function is confirmed by the excellent agreement between our results and those reported in the literature, which validates the adopted approach. The values of these coefficients were determined with oxygen partial pressure in capillary equal to 100 mmHg (healthy tissue) and 50 mmHg (tumor tissue). These pressures were also adopted in the current work as the value of *P_b,inlet_* in Equation (13) [5].

Figure 4d shows the initial distribution of *c*_3O2_ obtained for the data in Table 4 and the random *M*_0_ data, while Figure 4a shows the capillaries for which results related to the partial pressure and time-varying parameters will be presented. As can be seen, they lie near the external boundary of tissue Γ_0_, close to the optic axis of the laser beam, and represent all capillary types adopted in the simulation.

**Figure 6:**
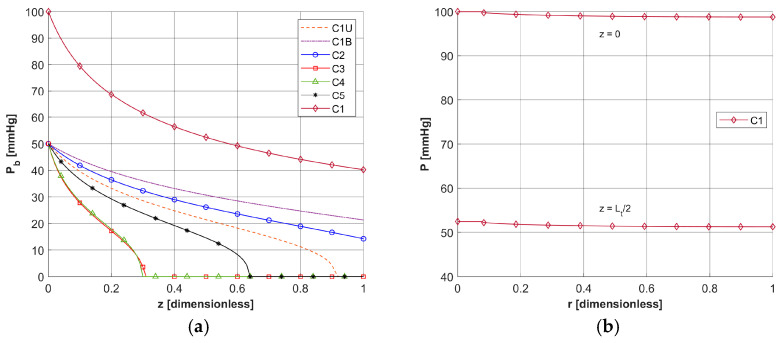
Distribution of the oxygen partial pressure for selected capillaries from the tumor subdomain in the axial direction (**a**) and in the radial direction for a C1-type capillary from the healthy tissue subdomain (**b**).

Figure 5 and Figure 6 show the results obtained for the Krogh cylinder model (12)–(13) for the capillaries marked in Figure 4a and for the capillary from the healthy tissue subdomain. Since the capillaries dimensions are different (see Table 4), the coordinates were divided by the corresponding capillary dimension to compare the results (for the radial direction, they were divided by *R_t_* and for the axial direction, they were divided by *L_t_*).

**Figure 7:**
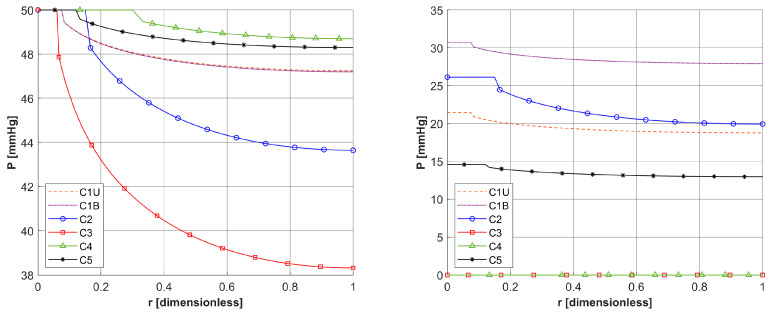
Distribution of the oxygen partial pressure in radial direction for selected capillaries from the tumor subdomain for *z* = 0 and *z* = *L_t_*/2.

In Figure 6a, the distributions of oxygen partial pressure for selected capillaries from the tumor subdomain and capillary type C1 from the healthy tissue subdomain in the axial direction are shown. It can be seen that most of the capillaries of the tumor subdomain (C1U, C3, C4, C5) show hypoxia (see Figure 4d). In the case of the capillaries C3 and C4, it occurs already before half of their lengths. In contrast, the *P_b_* value at the capillary outlet for C1 is about 40 mmHg and is consistent with values achieved for similarly configured models, for example, in [16]. The distributions in the radial direction (Figure 6b and Figure 7) show a pressure drop at the capillary-tissue boundary due to resistance during the diffusion of oxygen to the tissue subdomain, but in all the cases shown it is not large (up to 3 mmHg). For the capillaries highlighted in Figure 4, the values of *c*_3O2,*init*_ were as follows: C1U: 2.6 × 10^−8^ [mol cm^−3^], C1B: 3.9 × 10^−8^ [mol cm^−3^], C2: 2.9 × 10^−8^ [mol cm^−3^], C3: 0.6 × 10^−8^ [mol cm^−3^], C4: 0.9 × 10^−8^ [mol cm^−3^], C5: 1.9 × 10^−8^ [mol cm^−3^].

### 3.2. Bioheat Transfer and PDT Reactions Models

**Figure 8:**
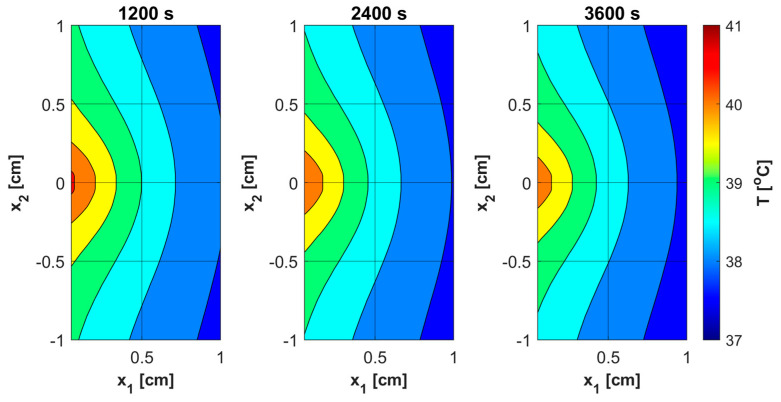
Distributions of temperature.

As already mentioned, PDT treatment does not involve large temperature increases (Figure 8). Table 5 collects results on the maximum temperature values, the Arrhenius integral *Arr*, the damage-dependent perfusion coefficient *w*(*Arr*), and the effective scattering coefficient μ′*_s_*(*Arr*). For all parameters except temperature, the highest values were recorded at the end of the simulation, that is, for time *t* = 3600 s. In the case of the Arrhenius integral, values are reached that for the function of perfusion coefficient correspond only to a stage of vasodilatation (cf. Equation (7)). The situation is similar for the effective scattering coefficient (cf. Equation (6)). For temperature, the highest values are reached for 892–907 s, followed later by a mild decrease due to the cooling effect resulting from increased perfusion. Therefore, Table 5 gives the maximum and final temperature values for each capillary.

**Figure 9:**
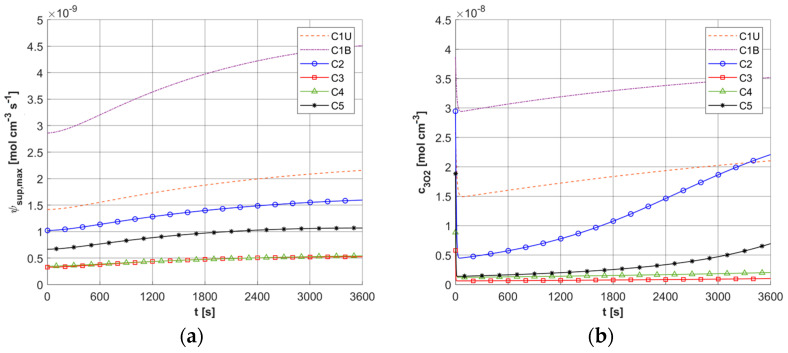
Histories of maximum oxygen supply ψ*_sup,max_* (**a**) and *c*_3O2_ concentration (**b**).

**Figure 10:**
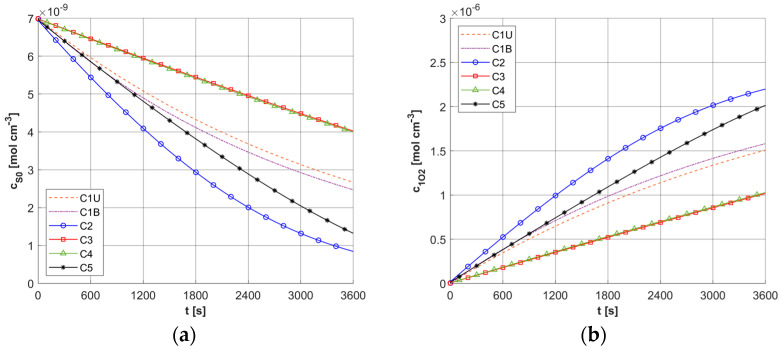
Histories of *c*_S0_ (**a**) and *c*_1O2_ concentrations (**b**).

Figure 9a shows the history of ψ*_sup,max_* estimated on the base of Formula (11). Note that this relationship combines the variable blood velocity *u_b_* resulting from the variable perfusion (cf. Equation (10)), the geometric parameters of the capillaries (*R_c_*, *R_t_*, and *L_t_*), and the random value *M*_0_. The value of ψ*_sup,max_* affects the other remaining dependent variables of the PDT model, namely the concentrations of triplet oxygen *c*_3O2_ (Figure 9b), photosensitizer *c*_S0_ (Figure 10a), and singlet oxygen *c*_1O2_ (Figure 10b). At the beginning of the simulation, the rapid drop in *c*_3O2_, when photosensitizer concentration is highest, is very evident (Figure 9b). It can also be seen that in capillaries showing hypoxia (C4, C3, C5, C1U) (cf. Figure 6), the return to the initial concentration of ^3^O_2_ is the slowest. Capillary C1B, which for *t* = 0 s had the highest *c*_3O2_, has the highest ψ*_sup,max_* values throughout the simulation, and for time *t* = 3600 s, it was closest to reaching the initial value of *c*_3O2_. In Figure 10, it is obvious that for hypoxic capillaries C4 and C3, the slowest photosensitizer burn reaction occurs, and consequently the smallest values of *c*_1O2_ are produced. The fastest reactions occur for capillaries C2 and C5, and only the latter show hypoxia.

**Figure 11:**
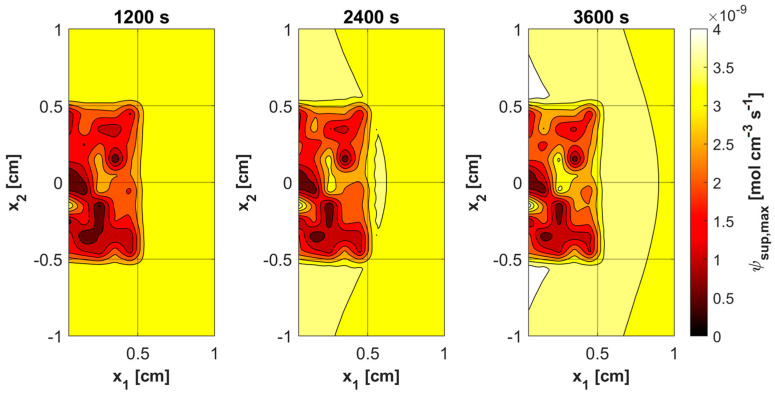
Distributions of maximum supply rate ψ*_sup,max_*.

**Figure 12:**
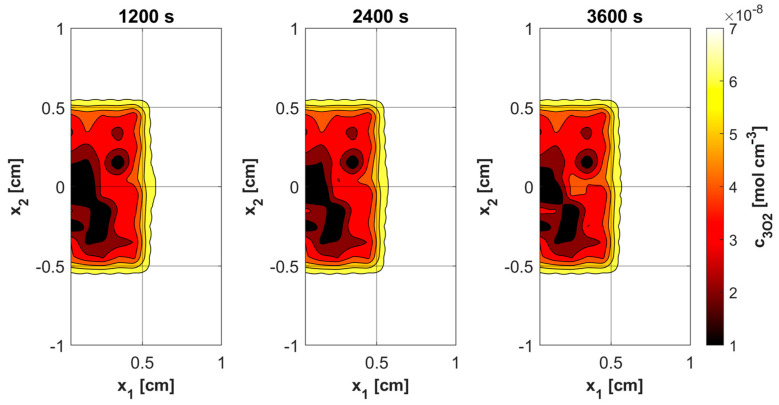
Distributions of ^3^O_2_ concentration.

**Figure 13:**
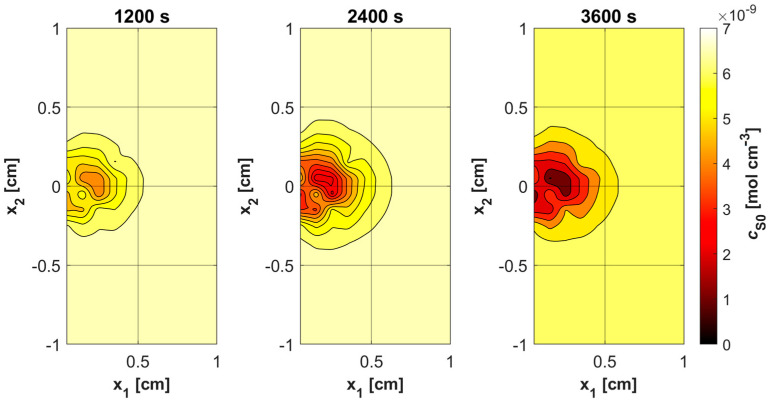
Distributions of S_0_ concentration.

**Figure 14:**
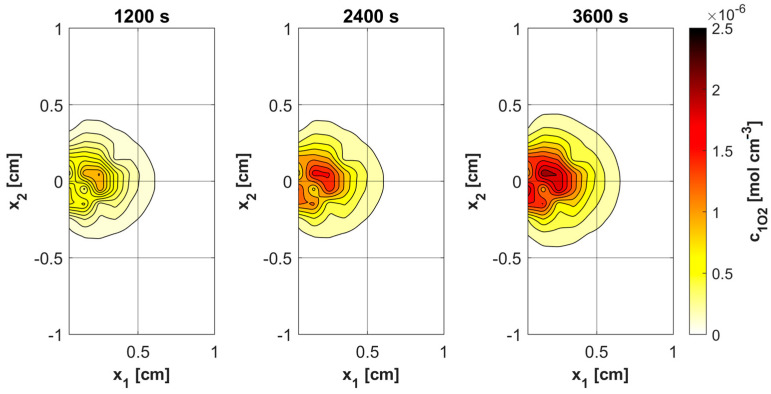
Distributions of ^1^O_2_ concentration.

Figure 11, Figure 12, Figure 13 and Figure 14 show the distribution of PDT-related parameters for times 1200, 2400, and 3600 s. It is visible that, due to the diverse structure of the tumor subdomain, they are irregular. It is worth noting that for triplet oxygen concentration, the changes are limited only to the tumor subdomain, while in the area of healthy tissue during the whole simulation, the value of *c*_3O2_ is constant. For *c*_S0_ and *c*_1O2_, the areas where the irregularities occur are associated with laser energy deposition. For photosensitizer concentration, a steady decrease is also visible in whole domain under consideration, which is consistent with natural processes in tissue.

### 3.3. The Tortuosity Coefficient

The models of bioheat transfer and PDT reactions considered in the current work are linked through the perfusion coefficient—the blood velocity in the capillary relationship. The calculated blood velocity in the capillary on the basis of the varying perfusion coefficient, taking into account an additional coefficient related to capillary tortuosity (Equation (10)), is then used in the model to determine the initial concentration of *c*_3O2_ (13)–(14) and in the relationship to determine ψ*_sup,max_* (Equation (11)). The purpose of building a model of this type is to provide information about the concentration ranges of parameters associated with the PDT model, which can ultimately assist the treatment preparation process, in a way that makes it more effective. The latter is especially true because the diverse structure of the tumor area has been taken into account here. Blood velocity in the capillary, as a parameter related to oxygen transfer to tissues and resulting from the bioheat transfer model, is quite a key parameter from this point of view.

The blood velocity in the capillary was analyzed to determine the ranges of ψ*_sup,max_*, *c*_3O2_, *c*_S0_, and *c*_1O2_ values achieved during the simulation of the PDT process. Since in the current work *u_b_* was disturbed by a random factor related to capillary tortuosity, three variants were considered:

- Variant 1: the tortuosity coefficient was not considered in the calculation.
- Variant 2: the tortuosity coefficient was considered only during the calculation of initial triplet oxygen concentration.
- Variant 3: the tortuosity coefficient was taken into account both during the calculation of the initial triplet oxygen concentration and during the simulation. This is the variant for which the calculations are presented in this work.

Comparisons were made in Table 6, Table 7, Table 8 and Table 9, for times of 1800 and 3600 s, additionally determining the difference between the largest and smallest values for each case. For the maximum supply rate ψ*_sup,max_* and the concentration of *c*_3O2_, the greatest differences were observed for variant 3, which is due to the direct link between these parameters and the blood velocity in the capillary (for ψ*_sup,max_* by Formula (11) throughout the simulation, for *c*_3O2_ via initial triplet oxygen concentration). It is slightly different for *c*_S0_ and *c*_1O2_ concentrations. For a time of 1800 s, the biggest differences are found for variant 1, while for *t* = 3600 s similarly as before for variant 3.

### 3.4. Results Verification

The results obtained were verified on the results available from the literature and analytical solutions.

#### 3.4.1. Laser Energy Deposition and Bioheat Transfer

For the task of estimating the temperature field in biological tissue exposed to a laser impulse, calculations were carried out using the broad beam laser method (BBL), in which [25]:

(28) $$Q_{las}(x_{1},t)=\mu_{a}\phi_{0}\left[c_{1}^{*}\exp\left(-k_{1}^{*}\frac{x_{1}}{\delta_{opt}}\right)-c_{2}^{*}\exp\left(-k_{2}^{*}\frac{x_{1}}{\delta_{opt}}\right)\right]$$

where

(29) $$\delta_{opt}=\frac{1}{\sqrt{3\mu_{a}\left[\mu_{a}+\mu_{s}(1-g)\right]}}$$

is the effective penetration depth, while coefficients $c_{1}^{*},c_{2}^{*},\;k_{1}^{*}$, and $k_{2}^{*}$ are determined by Monte Carlo solutions [25,49]. The diffuse reflectance was assumed to be *R_d_* = 0.05, and then the coefficients were calculated on the basis of the following formulas:

(30) $$c_{1}^{*}=3.09+5.44R_{d}-2.12\exp(-21.5R_{d})$$

(31) $$c_{2}^{*}=2.09-1.47R_{d}-2.12\exp(-21.5R_{d})$$

(32) $$k_{1}^{*}=1-\left(1-\frac{1}{\sqrt{3}}\right)\exp(-20.1R_{d})$$

(33) $$k_{2}^{*}=1.66\exp(3.4R_{d})$$

**Figure 15:**
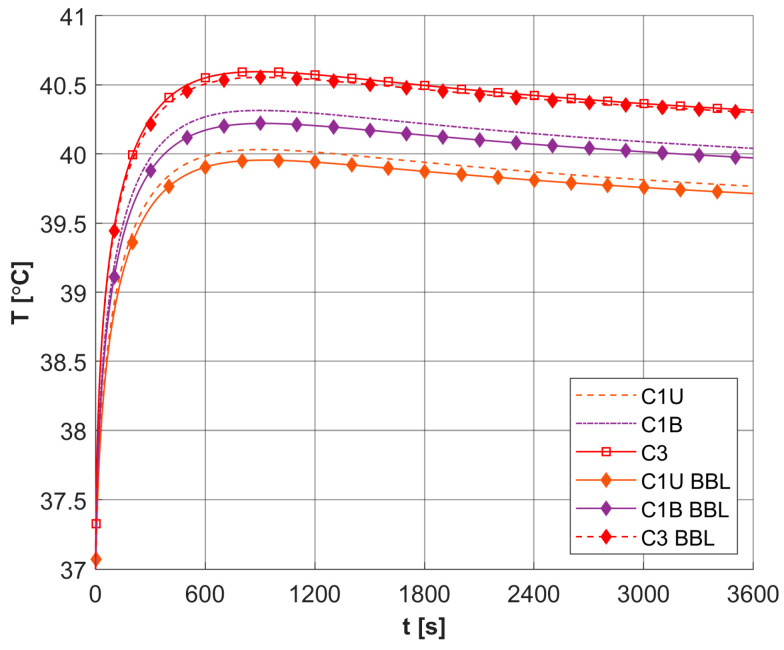
Comparison of temperature history in selected capillaries for models using different descriptions of laser heat source function (BBL, broad beam laser method, other curves, optical diffusion equation; relative errors: C1U: 0.166%, C1B: 0.207%, C3: 0.076%).

Figure 15 shows a comparison of the results obtained for three selected capillaries C1U, C1B, and C3 for models using the optical diffusion model (Equation (3)) and BBL approach (Equation (28)). It can be seen that the differences between the results are small. Additionally, a partial comparison was made with the results presented in [25] for the 1D task for highly scattering tissue. At this stage, the thermophysical and optical parameters, as well as boundary conditions from [25], were adopted, while the dimensions of the boundary and internal elements used in the BEM discretization were chosen to be similar to the discretization in [25]. The maximum temperature obtained at the boundary Γ_0_ for ϕ_0_ = 3 × 10^4^ [mW cm^−2^] and *t_exp_* = 5 s was analyzed. A value of 54.87 °C was obtained for the model using BBL and 57.52 °C for the model using the optical diffusion equation, which is not very different from the 55.7 °C obtained in [25].

#### 3.4.2. Krogh Cylinder Model

The results for the oxygen distribution model (radial) were compared with the solutions presented in [16] for a similar model. For these reasons, the data contained in [16] were adopted with *R_c_* = 2.5 [μm], *R_t_* = 25 [μm], and *L_t_* = 500 [μm]. Additionally, the current model was verified using of the analytical solution that exists for *P_crit_* = 0 in the form:

(34) $$P_{t}(r)=P_{b}+\frac{M_{0}}{2K_{t}}\left[\frac{1}{2}\left(r^{2}-R_{c}^{2}\right)-R_{t}^{2}\ln\frac{r}{R_{c}}\right]+\frac{M_{0}\pi }{k}\left(R_{c}^{2}-R_{t}^{2}\right)$$

**Figure 16:**
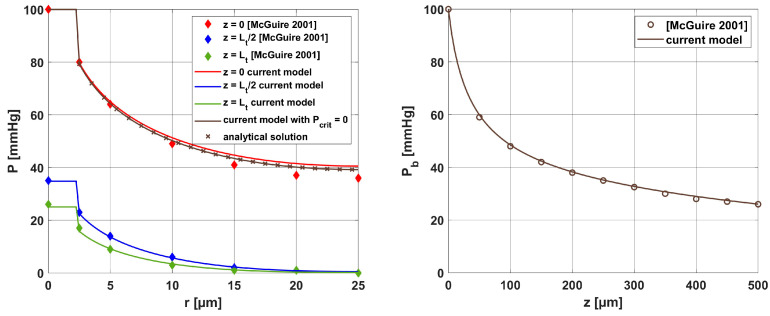
Comparison of the results for the Krogh cylinder model of the current work with the analytical solution and the results of [16] (relative errors for radial direction: *z* = 0: 4.726%, *z* = *L_t_*/2: 1.697%, *z* = *L_t_*: 5.392%; *P_crit_* = 0 case: 0.004%; relative error for axial direction: 1.110%).

All results were collected in Figure 16, with comparisons for the analytical solution (34) at *z* = 0. It can be seen that the results are similar for most curves. Only small differences were observed for comparison with [16] near the capillary inlet, for *r* = *R_t_*.

#### 3.4.3. PDT Reactions Model

To the best of our knowledge, there are currently no reference results for the combined bioheat and PDT models. Therefore, the results from the current PDT model were related to the results in which the influence of thermal phenomena on the photochemical reactions taking place was not taken into account.

The paper [5] presents the results of the analysis of the PDT model for different values of maximum oxygen supply ψ*_sup,max_*, with this parameter estimated based on the Krogh cylinder model, for a wide range of geometric parameters and the rate of oxygen consumption *M*_0_. Simulations were conducted for different values of laser surface irradiance ϕ_0_, the highest being 150 [mW cm^−2^]. For this value of ϕ_0_, calculations were carried out to compare the current model with the results presented in [5].

**Figure 17:**
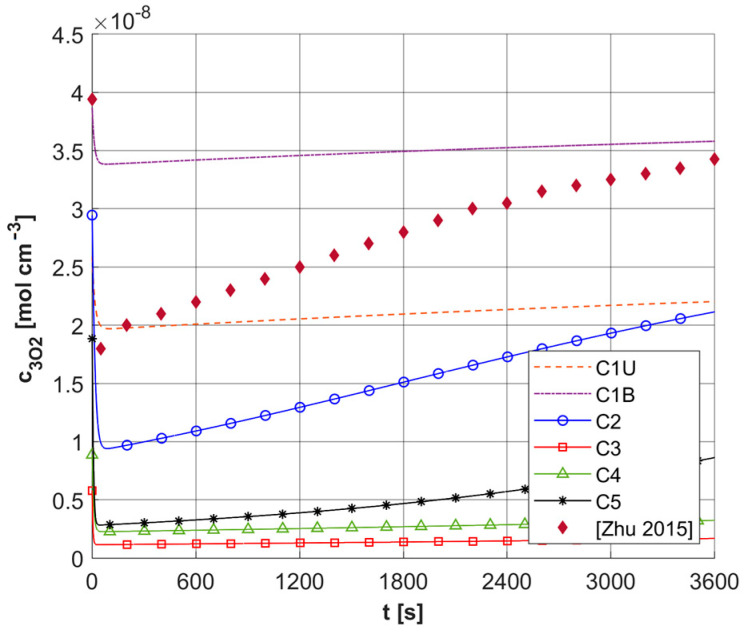
Comparison of the results of the current model for ϕ_0_ = 150 [mW cm^−2^] with the results presented in [5].

First, a comparison was made for a task using an identical configuration, as shown in the Section 3. The results are shown in Figure 17. As can be seen, the results of [5] are within the range of results achieved for the current model, while it should be added that [5] used a constant value of *c*_3O2,*init*_ = 3.941 × 10^−8^ [mol cm^−3^], and in addition, the curve presented was defined as referring to the average values of triplet oxygen concentration *c*_3O2_. The sharp drop in *c*_3O2_ at the beginning of the simulation is clearly visible.

**Figure 18:**
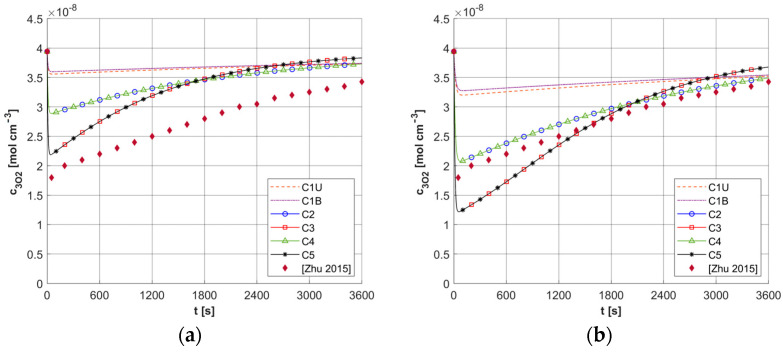
Comparison of results for fixed values of ψ*_sup,max_* = 4.2 × 10^−9^ [mol cm^−3^ s^−1^] (**a**) and 2.1 × 10^−9^ [mol cm^−3^ s^−1^] (**b**) and fixed *c*_3O2,*init*_ with the results of [5] (ϕ_0_ = 150 [mW cm^−2^]).

As mentioned, the results of ψ*_sup,max_* calculations for a number of different capillary configurations were presented in [5]. Figure 18 shows the *c*_3O2_ histories obtained for the current model for two constant values of ψ*_sup,max_*, that is, for 4.2 × 10^−9^ and 2.1 × 10^−9^ [mol cm^−3^ s^−1^]. These values were obtained for a capillary with geometric dimensions like the C3 capillary (see Table 4) and constant values of *u_b_* = 0.01 and 0.005 [cm s^−1^] and constant value *M*_0_ = 2.4 × 10^−9^ [mol cm^−3^ s^−1^]. Furthermore, a constant value of *c*_3O2,*init*_ was assumed for the current model, identical to that in [5]. As can be seen in the presented calculation cases, the results obtained from the current model, using a pattern that takes into account differentiated capillaries in the tumor area, are in the vicinity of the reference results. Any differences can, of course, be explained by the differences between the current model and the model presented in [5], most notably the link between the current model and the bioheat transfer task.

## 4. Discussion

The analysis of the combined bioheat transfer and PDT reactions models carried out in this study captured some of the characteristics of the tumor tissue that result from the atypical vascular pattern. This was achieved by adopting different geometric parameters of capillaries in the subdomain of tumor and varying values of parameters related to the PDT model, i.e., oxygen consumption rate, blood velocity in capillary, and initial distribution of triplet oxygen. The latter parameter was calculated on an additional model in the form of a Krogh cylinder, while the capillary blood velocity was derived from the thermal damage-dependent perfusion coefficient. From the point of view of the assumed Function (7), a state of increased perfusion was achieved. As the calculation results for the assumed laser pulse value show, there are small temperature increases, up to about 40.5 °C (Table 5), which do not allow high thermal damage values to be reached. The damage-dependent value of the effective scattering coefficient (6) changed little.

To further differentiate the parameters, a random coefficient related to blood velocity in capillary was introduced to express the tortuosity of capillaries, i.e., their abnormal shape in the tumor area. As the simulations showed, this factor quite strongly influenced the range of values obtained during simulations (Table 6) for maximum supply rate, and thus the concentration of ^3^O_2_ and ^1^O_2_ (Table 7 and Table 9). It should be noted that the results presented in Table 6, Table 7, Table 8 and Table 9 concern only six selected capillaries in the tumor area, and due to the randomness of the parameters in this area, the ranges of the values of the mentioned parameters are quite larger.

As mentioned, the bioheat and PDT models have so far been considered separately, so the necessary values of blood velocity in the capillary were taken as constant. Studies in [10] show that this can correspond to a fairly wide range of maximum supply rate ψ*_sup,max_*, with values derived from the associated models yielding results outside the range obtained for constant blood velocity in capillary *u_b_*. It is worth mentioning that the adopted value of the initial perfusion coefficient (*w*_0_ = 0.00125 s^−1^) is one of several values prevalent in the literature for soft tissues in bioheat transfer tasks [18,19,29,39,40]. Adopting a coefficient value more similar to the tissue type under consideration could bring these ranges closer, while introducing a tortuosity coefficient potentially widens the ranges.

Other parameters included in the PDT model were also generally assumed to be fixed. This refers to the oxygen consumption rate *M*_0_, or constant light energy deposition. This makes it difficult to compare the current model with them, but in a previous work [9] it was shown that, taking the relevant parameters as constants, the model presented gave results consistent with those known from the literature [50]. Similarly, the model used to calculate the initial triplet oxygen concentration (13) and (14) produces results consistent with those existing in the literature for the distribution of the partial pressure in the Krogh cylinder [16], as well as similar to the values assumed for the *c*_3O2,*init*_ [5]. It is worth mentioning here that due to the use of this model only for the purpose of determining the initial conditions for the PDT task, the Bohr effect, i.e., the variation of the parameters of the oxyhemoglobin dissociation curve under the influence of temperature, was not considered here [11,51,52].

The results presented here show that an appropriate model of the tumor subdomain structure makes it possible to reproduce phenomena within it, such as nonuniform concentration of triplet oxygen and associated areas of hypoxia, which can affect the final course of reactions that occur during PDT. It should be noted that there are quite a few parameters in the PDT model, and it would be worthwhile to perform a sensitivity analysis of this model. A particularly interesting approach here seems to be the direct method in which the sensitivity functions of the parameters for which the sensitivity analysis is performed are determined [22,53,54]. In addition to such important parameters as blood velocity in capillary, oxygen consumption rate, or initial concentration of triplet oxygen and photosensitizer, it can refer to photochemical parameters occurring in the PDT model, which are mainly related to the type of photosensitizer used. This could help select them for a specific treatment or aid in the design of new types of photosensitizers by indicating their desirable characteristics.

A Krogh-cylinder-based model was applied to determine the initial concentration of triplet oxygen. The element of this model is the oxyhemoglobin curve, for which the Hill model was adopted in the paper (Equation (14)). As mentioned above, the shape of the curve is influenced by temperature, pH, concentrations of carbon dioxide, and 2,3-DPG (2,3-diphosphoglycerate). The values of some of these parameters for tumor tissue are different from those for healthy tissue; for example, tumor tissue is more acidic and there is a higher concentration of CO_2_, which is at least partly due to an abnormal vascular pattern in the tumor area. There are ODC models in the literature to account for changes in these parameters, and it would be advisable to use them in future as well [55,56].

Various sources of light, e.g., linear once, are used in actual PDT procedures, while in the current work it was a laser beam. This was clearly related to areas of change in photosensitizer and singlet oxygen concentration (Figure 13 and Figure 14), while for maximum supply rate and triplet oxygen concentration changes occur throughout the tumor subdomain and are more related to variable blood velocity in the capillary (Figure 11 and Figure 12). It is clear that adopting a different type of light source would affect the mentioned characteristics [3,50].

One direction for the development of the presented model will certainly be to use a bioheat transfer model different from the Pennes formula. It is still probably the most popular bioheat transfer equation, although its drawbacks are widely described and discussed in the literature. The most recent developments in this field involve models based on the porous bodies theory, known as the dual-phase lag equation. They allow calculation of propagation delays in both temperature and heat flux, and one of the main parameters of such an equation is porosity, which describes blood vessels in the tissue area density. From this point of view, the connection between the DPL equation and the proposed way of modeling the tumor area seems interesting [20,22,30,31,33].

The final conclusion of the presented research is certainly that even small increases in the temperature of biological tissue from what occurs during PDT treatments should be taken into account as a potential factor affecting tissue processes. This type of relationship has already been reported in various cases of experimental immunology, where the effect of temperature on the dynamics of the tumor environment and the regulation of the immune response has been demonstrated.

## Figures and Tables

**Table 1:** Parameters for bioheat transfer, tissue damage, and laser energy deposition model.

Bioheat Transfer Model			Tissue Damage Model		
Parameter/Unit	Value	Reference	Parameter/Unit	Value	Reference
λ [W m^−1^ K^−1^]	0.75	[10]	*A* [s^−1^]	1.98 × 10^106^	[10,18]
*c* [MJ m^−3^ K^−1^]	3	[10]	*E* [J mol^−1^]	6.67 × 10^5^	[10,18]
*w*_0_ [s^−1^]	0.00125	[10]	*R* [J mol^−1^ K^−1^]	8.314	[10,18,29]
*Q_met_* [W m^−3^]	250	[10]	**Laser energy deposition model**		
*c_B_* [MJ m^−3^ K^−1^]	3.9962	[10]	**Parameter/Unit**	**Value**	**Reference**
*T_B_* [°C]	37	[10]	μ*_a_* [cm^−1^]	1.03	[10]
α [W m^−2^ K^−1^]	10	[10]	μ′*_s nat_* [cm^−1^]	13.46	[10]
*T_amb_* [°C]	20	[10]	μ′*_s den_* [cm^−1^]	26.92	[10]
*T_init_* [°C]	37	[10]	ϕ_0_ [mW cm^−2^]	300	[10]
boundary elements	160		*r_beam_* [mm]	1	[10]
internal elements	1600		grid	40 × 40 nodes	

**Table 2:** Parameters for calculation of initial triplet oxygen concentration (Krogh cylinder model).

	Krogh Cylinder Model	
Parameter/Unit	Value	Reference
*K_t_* [(cm^2^ s^−1^)(mol cm^−3^ mmHg^−1^)]	2.202 × 10^−14^	[5,14]
*P_crit_* [mmHg]	1	[12,16,17]
*k* [(cm^2^ s^−1^)(mol cm^−3^ mmHg^−1^)]	2.79 × 10^−13^	[16,41]
κ*_b_* [mol cm^−3^_blood_]	9.1 × 10^−6^	[14]
*n*	2.57	[41]
*P*_50_ [mmHg]	27	[41]
α_3O2,*t*_ [mol cm^−3^ mmHg^−1^]	1.295 × 10^−9^	[5,14]

**Table 3:** Parameters for PDT reactions model.

	PDT Reactions Model	
Parameter/Unit	Value	Reference
δ [mol cm^−3^]	33 × 10^−9^	[5]
β [mol cm^−3^]	11.9 × 10^−9^	[5]
σ [cm^3^ mol^−1^]	7.6 × 10^4^	[5]
ξ [cm^2^ mW^−1^ s^−1^]	3.7 × 10^−3^	[5]
*c*_S0,*init*_ [mol cm^−3^]	7 × 10^−9^	[5]

**Table 4:** Types of capillaries applied in the abnormal vascular pattern model.

Capillary Type	*R_c_* [μm]	*R_t_* [μm]	*L_t_* [μm]
C1	2.5	30	400
C2	10	60	100
C3	4	60	220
C4	10	30	220
C5	4	30	100

**Table 5:** The maximal values of temperature, Arrhenius integral, perfusion coefficient, and effective scattering coefficient.

Capillary	*T_max_* [°C]	*T_end_* [°C]	*Arr_max_*	*w_max_* × 1000 [s^−1^]	μ′_s *max*_ [cm^−1^]
C1U	40.03	39.77	0.030	1.903	13.87
C1B	40.32	40.04	0.038	1.972	13.97
C2	40.23	39.96	0.036	1.954	13.94
C3	40.60	40.32	0.048	2.001	14.10
C4	40.23	39.96	0.036	1.954	13.94
C5	40.60	40.32	0.048	2.001	14.10

**Table 6:** The values of maximum oxygen supply rate ψ*_sup,max_* [mol cm^−3^ s^−1^] × 10^9^ for different variants of computations.

Capillary		1800 s			3600 s	
	Variant 1	Variant 2	Variant 3	Variant 1	Variant 2	Variant 3
C1U	3.79	3.79	1.88	4.35	4.35	2.16
C1B	3.97	3.97	3.97	4.51	4.51	4.51
C2	2.62	2.62	1.40	2.99	2.99	1.60
C3	3.17	3.17	0.48	3.49	3.49	0.52
C4	3.06	3.06	0.47	3.50	3.50	0.54
C5	3.70	3.70	0.97	4.07	4.07	1.07
max–min	1.36	1.36	3.50	1.53	1.53	3.99

**Table 7:** The values of the ^3^O_2_ concentration [mol cm^−3^] × 10^8^ for different variants of computations.

Capillary		1800 s			3600 s	
	Variant 1	Variant 2	Variant 3	Variant 1	Variant 2	Variant 3
C1U	3.29	2.21	1.84	3.55	2.35	2.10
C1B	3.29	3.29	3.29	3.52	3.52	3.52
C2	2.68	2.04	1.08	3.58	2.71	2.21
C3	2.33	0.43	0.08	2.96	0.52	0.10
C4	3.10	0.68	0.15	3.89	0.79	0.20
C5	3.73	1.51	0.25	4.34	1.84	0.70
max–min	1.40	2.87	3.22	1.38	3.00	3.42

**Table 8:** The values of the *S*_0_ concentration [mol cm^−3^] × 10^9^ for different variants of computations.

Capillary		1800 s			3600 s	
	Variant 1	Variant 2	Variant 3	Variant 1	Variant 2	Variant 3
C1U	3.88	4.14	4.33	2.20	2.50	2.67
C1B	4.12	4.12	4.12	2.47	2.47	2.47
C2	1.79	2.00	2.94	0.38	0.48	0.84
C3	0.86	2.59	5.45	0.06	0.82	4.02
C4	1.62	3.17	5.42	0.33	1.37	3.99
C5	0.56	1.06	3.82	0.03	0.11	1.32
max–min	3.55	3.08	2.51	2.44	2.39	3.18

**Table 9:** The values of ^1^O_2_ concentration [mol cm^−3^] × 10^6^ for different variants of computations.

Capillary		1800 s			3600 s	
	Variant 1	Variant 2	Variant 3	Variant 1	Variant 2	Variant 3
C1U	1.07	0.98	0.91	1.68	1.57	1.51
C1B	0.98	0.98	0.98	1.58	1.58	1.58
C2	1.84	1.76	1.41	2.38	2.34	2.20
C3	2.19	1.54	0.52	2.51	2.21	1.02
C4	1.90	1.32	0.53	2.40	2.00	1.03
C5	2.31	2.11	1.09	2.52	2.49	2.01
max–min	1.32	1.14	0.89	0.94	0.92	1.18

## Data Availability

The original contributions presented in this study are included in the article. Further inquiries can be directed to the corresponding author.
